# Epistasis and the Evolution of Antimicrobial Resistance

**DOI:** 10.3389/fmicb.2017.00246

**Published:** 2017-02-17

**Authors:** Alex Wong

**Affiliations:** Department of Biology, Carleton University, OttawaON, Canada

**Keywords:** antimicrobial resistance, genetic interaction, epistasis, compensatory evolution, multi-drug resistance

## Abstract

The fitness effects of a mutation can depend, sometimes dramatically, on genetic background; this phenomenon is often referred to as “epistasis.” Epistasis can have important practical consequences in the context of antimicrobial resistance (AMR). For example, genetic background plays an important role in determining the costs of resistance, and hence in whether resistance will persist in the absence of antibiotic pressure. Furthermore, interactions between resistance mutations can have important implications for the evolution of multi-drug resistance. I argue that there is a need to better characterize the extent and nature of epistasis for mutations and horizontally transferred elements conferring AMR, particularly in clinical contexts. Furthermore, I suggest that epistasis should be an important consideration in attempts to slow or limit the evolution of AMR.

## Introduction

The same mutation, occurring in two different individuals, can have drastically different consequences for phenotype and for fitness. A mutation’s effects may depend on the environment in which an individual finds itself, or on the genetic background on which the mutation occurs. In a classic example familiar from undergraduate genetics textbooks, the *bw* mutation causes a brown eye phenotype in wild-type *Drosophila melanogaster*. However, the same *bw* mutation occurring on a *cn* background (where the *cn* mutation usually causes a cinnabar eye color), results in a white-eye phenotype. The microbial literature also abounds with examples of the importance of genetic background. In any toxin-antitoxin system, the phenotypic effects of the presence of the toxin-encoding gene are entirely dependent on genetic background: the toxin gene is lethal when the antitoxin-encoding gene is absent, but not when it is present. The influences of genetic background on a mutation’s effects are often referred to as “genetic interactions” or “epistasis,” and can have profound implications for organismal fitness.

**FIGURE 1:**
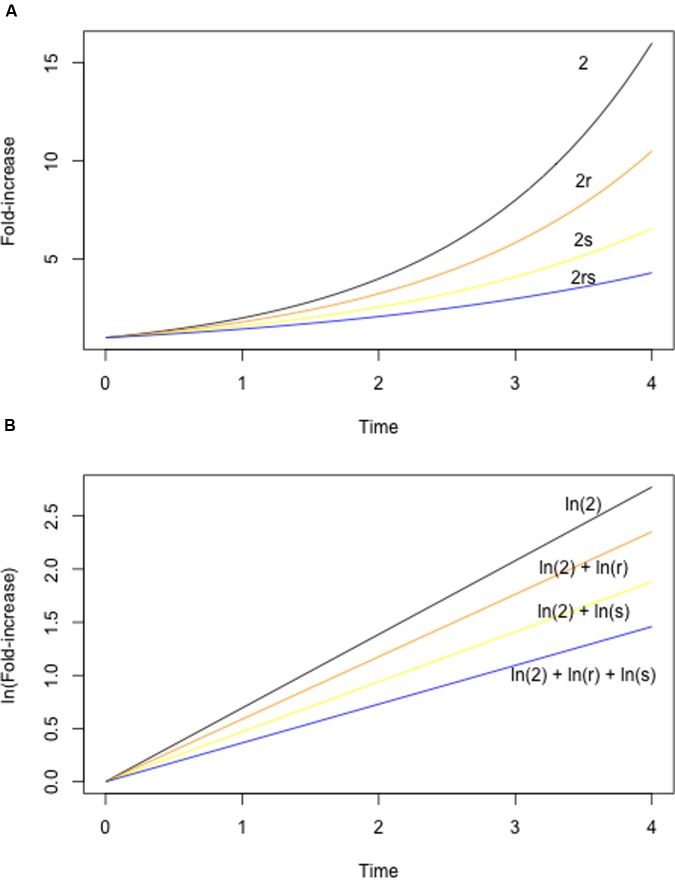
**Null models for epistasis.** The expected fitness of a double mutant can be calculated using either a multiplicative **(A)** or additive **(B)** model. A multiplicative model is appropriate under a standard exponential growth model, with population size increasing according to λ^t^ (where t is time). **(A)** Shows exponential growth curves for a hypothetical wild-type (black, λ = 2, indicating that population size doubles with each unit time), two mutants (orange, yellow, with λ reduced by a factor of *r* and *s*, respectively), and the double mutant (blue). Note that the growth parameter λ of the double mutant is determined by the *product* of *r* and *s.* An additive model is appropriate when population sizes are log-transformed, yielding a linear relationship between ln(population size) and time. **(B)** Shows log-transformed growth curves for the same mutants as in **(A)**. Note that the fitness of the double mutant is now determined by ln(*r*) + ln(*s*), where the log of the population size at time t is given by e^In(λ*t*)^.

The study of epistasis has a long history in both theoretical and experimental biology (e.g., Bateson, 1909; Wright, 1968; Cordell, 2002; Weinreich et al., 2005; Phillips, 2008). As a consequence, there is a great deal of diversity – and sometimes confusion – with respect to the terminology used to describe epistasis. In the current context, it is useful to consider epistasis in terms of the fitness effects of two mutations alone, or in combination. Fitness is an organism’s ability to survive and reproduce in a given environment, and in microbes fitness is often measured using competitive assays (Melnyk et al., 2015), exponential growth rates in liquid culture, or colony sizes on solid media. Formally, epistasis for fitness is defined as any situation in which the fitness of a double mutant differs from what is expected given the fitness of the constituent single mutants. The expected fitness is either the sum or the product of the single mutant fitness values, depending on the underlying model (**Figure 1**; see also Wade et al., 2001; Chevin, 2011). In either case, epistasis is inferred if the realized fitness of the double mutant is either higher or lower than expected.

**FIGURE 2:**
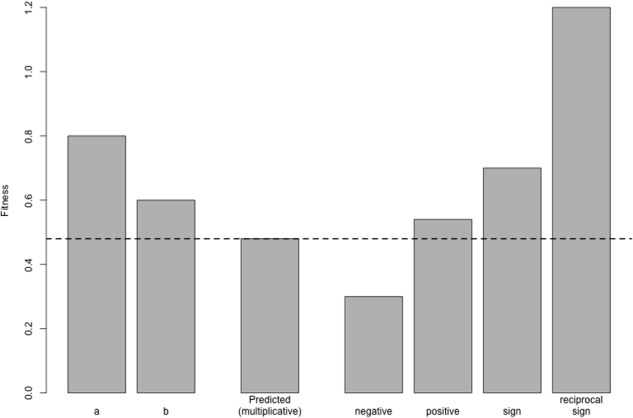
**Varieties of epistasis.** Here, two mutations *a* and *b* are individually deleterious with fitnesses of 0.8 and 0.6, respectively; the fitness of the wild-type is 1 by definition. In the absence of epistasis, these mutations combine *multiplicatively*, such that the fitness of the double mutant is the product of their individual fitness values (0.48, dotted line). Under *negative* epistasis, the fitness of the double mutant is lower than expected (here, 0.3). Under *positive* epistasis, double mutant fitness is greater than expected (0.54). The bar marked *sign* shows a situation whereby one mutation, *a*, changes its sign when paired with *b*: on the wild-type background, *a* is deleterious, but it is beneficial on the *b* mutant background (since the double mutant, with a fitness of 0.7, is more fit than the *b* mutant alone). Finally, in the case of *reciprocal sign* epistasis, both mutations switch their sign, becoming beneficial in combination with a fitness of 1.2. Note that both of these cases of sign epistasis are also cases of positive epistasis, since the double mutant fitness is higher than expected.

Amongst epistatic interactions, we can distinguish between three varieties (**Figure 2**): (1) *negative* epistasis, whereby the fitness of the double mutant is *lower* than expected, (2) *positive* epistasis, whereby the fitness of the double mutant is *higher* than expected, and (3) *sign* epistasis, whereby the sign of the fitness of a mutation changes depending on genetic background – here, a mutation may (for example) be deleterious on one genetic background, but may be beneficial or have no effect on a different genetic background (as is the case in toxin-antitoxin systems). *Reciprocal sign* epistasis refers to cases whereby both members of a pair of interacting mutations change their signs, such as when two individually deleterious mutations become beneficial in combination, or two individually beneficial mutations are deleterious in combination.

Large-scale screens in *Escherichia coli* (Butland et al., 2008; Typas et al., 2008; Babu et al., 2011, 2014) and yeasts (Roguev et al., 2007; Costanzo et al., 2010) have shown that epistasis is quite common. Such large-scale screens involve the construction of hundreds of thousands to millions of double knockout mutant combinations through the use of Hfr-mediated conjugation (*E. coli*) or mating (yeast). Babu et al. (2011), for example, investigated genetic interactions of genes involved in cell envelope biogenesis in *E. coli* by measuring the colony sizes of ∼350 000 double mutants. Remarkably, more than 54 000 genetic combinations were classified as epistatic in rich media, and more than 38 000 were considered epistatic in minimal media. These results suggest that epistasis is highly prevalent, such that the effects of a mutation may depend strongly on genetic background.

Epistasis has important implications for a wide range of evolutionary phenomena, including speciation (Wade, 2002; Presgraves, 2007), the evolution of sex (Charlesworth, 1990; Otto and Michalakis, 1998), the trajectory of evolution (Weinreich et al., 2006; Franke et al., 2011; Tenaillon et al., 2012), and much else besides (Wolf et al., 2000). Here, I discuss some of the implications of epistasis for the evolution and management of antimicrobial resistance (AMR), with a primary focus on pathogenic bacteria. Due to limitations in the published literature, most of the examples used here refer to chromosomal resistance mutations, rather than resistance elements harbored on mobile genetic elements (MGEs) like plasmids and integrons. Nonetheless, so far as data are available, many of the lessons learned from chromosomal resistance mutations also apply to MGEs; relevant examples are highlighted throughout. Because of its potential importance, I suggest that epistasis should be given greater consideration in the study of AMR. I argue that epistasis may have important roles to play in determining the trajectory of resistance evolution, in the persistence of costly resistance mutations, and in the evolution of multi-drug resistance (MDR). More positively, I suggest that we may in some instances be able to leverage epistasis in efforts to counteract AMR.

## The Evolutionary Trajectory of Resistance Acquisition

Epistatic interactions can have important consequences for the trajectory and tempo of adaptive evolution – that is, on what mutations accrue during adaptation, in what order, and how quickly. While much work in this area has not focused on AMR, there are a number of studies in AMR that do address these issues.

In laboratory selection experiments, it is often observed that fitness initially increases very rapidly, but that the rate of fitness increase drops over time – later on during a selection experiment, fitness increases at a much lower rate than during the early stages (e.g., Elena and Lenski, 2003; Barrick et al., 2009; see also Couce and Tenaillon, 2015). Several studies have shown that negative epistasis between beneficial mutations underlies this pattern – it appears that adaptive mutations, when combined, tend to have a smaller beneficial effect than would be expected (Chou et al., 2011; Khan et al., 2011; Schoustra et al., 2016); this pattern is referred to as “diminishing returns epistasis.” Under diminishing returns epistasis, an adapting population may accrue beneficial mutations at a fairly steady rate even while fitness improves by ever smaller steps (Barrick et al., 2009). Diminishing returns epistasis suggests a clear prediction regarding the evolution of AMR: when a bacterial population encounters a new drug, we would expect the bulk of adaptation to occur early on, with lower rates of adaptation as time passes. This prediction has been confirmed experimentally for adaptation of *Pseudomonas aeruginosa* to rifampicin (MacLean et al., 2010).

An additional consequence of epistasis – particularly sign epistasis – is that it can limit the number of evolutionary paths available to a population. Sign epistasis between mutations that are individually advantageous may have profound effects on the trajectory of evolution – if certain combinations of advantageous mutations are deleterious, then we do not expect to see these combinations persist. Such sign epistasis can constrain the number of routes available to adaptation. The evolution of classical β-lactamases into extended-spectrum β-lactamases (ESBLs), capable of degrading cephalosporins, serves as a striking example. Five mutations are required to convert a classical β-lactamase into an ESBL, such that there are in principle 120 possible routes to ESBL-activity. Weinreich et al. (2006) constructed all 32 possible double mutants, and found rampant sign epistasis, whereby many pairs of mutations showed decreased fitness (i.e., reduced ability to degrade cephalosporins). As a result, only 18 of the 120 possible evolutionary routes are viable, insofar as they do not involve reductions in fitness. A tempting conclusion, then, is that we might expect evolution to proceed in a repeatable manner – amongst the myriad of possible ways to evolve ESBL-activity, only a handful will be realized. However, many more evolutionary routes are available under sub-MIC drug concentrations (Mira et al., 2015), indicating that epistatic effects may be strongly environment-specific.

While the ESBL example suggests that epistasis can lead to evolutionary convergence, at least under specific conditions, sign epistasis can also lead to *divergence* between populations adapting to the same environments. Populations of *E. coli* adapting to high temperatures appear to adopt either of two mutually exclusive metabolic solutions, one involving mutations in RNA polymerase (*rpoBC)*, and the other involving mutations in the termination factor *rho* (Tenaillon et al., 2012). A number of mutations that arise frequently on *rpoBC* mutant backgrounds are never seen on *rho* mutant backgrounds, and vice versa, strongly suggesting sign epistasis. Thus, once a population adopts – by chance – either the *rho* or the *rpoBC-*mediated path to heat tolerance, it is committed to a particular evolutionary trajectory, and will diverge from populations adopting the alternative solution. There is little evidence for similar patterns in the context of AMR, however; several studies have suggested that populations adapting to antibiotics tend to converge on similar genetic solutions (Vogwill et al., 2014; Wong and Seguin, 2015).

## Epistasis and the Persistence of AMR Mutations

Many AMR mutations bear costs, insofar as they cause reduced fitness in the absence of antibiotic (Cong et al., 2007; Andersson and Hughes, 2010; Gerstein et al., 2012; Rosenthal, 2013; Vincent et al., 2013; Melnyk et al., 2015; Vogwill and MacLean, 2015). Melnyk et al. (2015) recently performed a meta-analysis of studies that estimated the fitness of strains bearing defined, chromosomal AMR mutations. Costs of resistance were widespread across drug classes and bacterial species, although data were sparse. In some cases (e.g., rifampicin, erythromycin), a single resistance mutation results in a ∼20% reduction in fitness under antibiotic-free laboratory conditions, while in others (e.g., trimethoprim), resistance is apparently cost-free. Fewer data are available for resistance elements harbored on MGEs. However, a number of studies have shown that carriage of plasmids or of other MGEs is often costly (Lee and Edlin, 1985; Lenski and Bouma, 1987; McDermott et al., 1993; Dahlberg and Chao, 2003; Enne et al., 2004a, 2005; Dionisio et al., 2005; Heuer et al., 2007; Marciano et al., 2007; Petersen et al., 2009; Starikova et al., 2012; Harms et al., 2013; Starikova et al., 2013; San Millan et al., 2014b). In a handful of cases, plasmid carriage has been shown to confer a benefit, either on naïve or clinical genetic backgrounds (Enne et al., 2004a), or in hosts that have co-evolved with a plasmid in the laboratory (Bouma and Lenski, 1988; Dionisio et al., 2005; Starikova et al., 2013). When plasmid carriage is costly, these costs are typically associated with an entire element (plasmid, transposon, etc…) potentially harboring many genes, and have not been ascribed to a specific resistance gene *per se*. However, costs associated with carriage of the non-conjugative pBR322 plasmid in *E. coli* are attributable to expression of its tetracycline resistance gene (Lee and Edlin, 1985; Valenzuela et al., 1996), and expression of the vancomycin resistance operon is required for costs of VanB-mediated resistance in *Enterococcus faecalis* (Foucault et al., 2010). More importantly, since transmissible resistance genes are in fact associated with larger elements, fitness of the entire element is arguably the more important parameter.

The widespread occurrence of costs of resistance points to the possibility of controlling resistance through cessation of use of a particular drug. For example, given that rifampicin resistance typically carries a cost, we might expect that resistance should decrease in frequency in clinical populations if use of rifampicin (and related compounds) were to be stopped. Interestingly, cessation of use has mixed results in mitigating AMR (Costelloe et al., 2010; Enne, 2010). Notably, in several cases, resistance has persisted for months or years despite a substantial reduction in prescription (Sundqvist et al., 2010).

**FIGURE 3:**
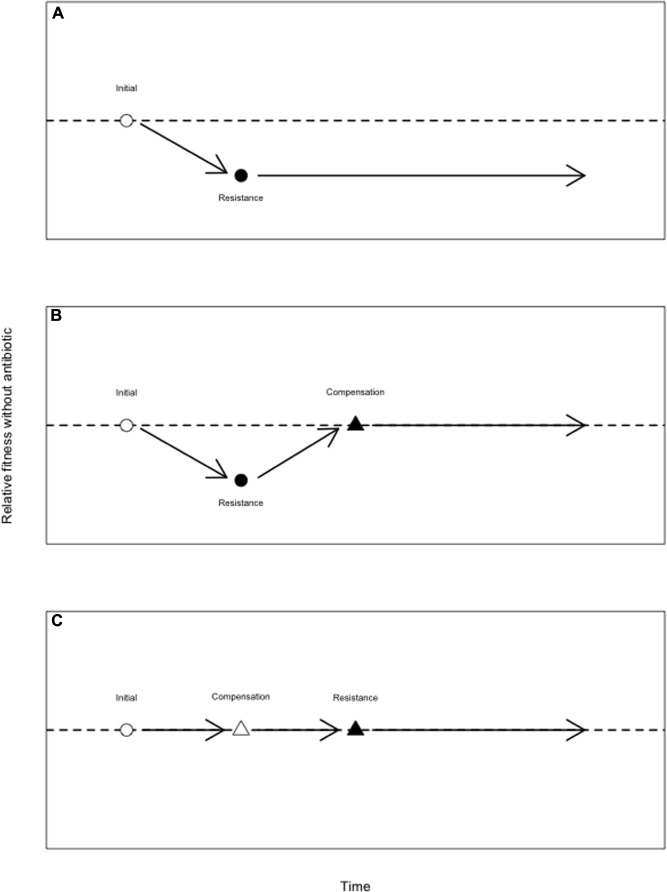
**Alleviation of the costs of antibiotic resistance by compensatory mutations.** **(A)** A drug susceptible genotype (open circle) experiences an antimicrobial resistance (AMR) mutation (closed circle), which reduces its fitness in the absence of antibiotic. Wild-type fitness is given by the dotted line. **(B)** A drug susceptible genotype (open circle) experiences an AMR mutation (closed circle), which reduces its fitness in the absence of antibiotic. This genotype subsequently sustains a compensatory mutation which restores fitness but does not eliminate resistance (close triangle). Here, a compensatory mutation arises after the costly AMR mutation. While the compensatory mutation is here depicted as neutral, such mutations may impose a fitness cost on the drug sensitive background (Schrag et al., 1997; Brandis et al., 2012). **(C)** Compensatory mutations that do not themselves carry a cost may also precede the AMR mutation. Here, a drug susceptible genotype experiences a mutation (open triangle) that does not affect fitness, but which later alleviates the fitness deficit that would have been imposed by the AMR mutation (closed triangle).

There are several possible explanations for the persistence of costly AMR alleles in the absence of antibiotic (Enne, 2010; Melnyk et al., 2015). Some AMR elements may be cost-free, either universally or in specific environments (Humphrey et al., 2012; Gifford et al., 2016), while AMR elements on plasmids may be maintained via co-selection for other plasmid-encoded functions (Pal et al., 2015). When an AMR mutation is initially costly, those costs may be reduced or eliminated by *compensatory mutations* – that is, mutations that increase fitness without eliminating resistance. The term “compensatory” is sometimes reserved for fitness-restoring mutations that occur after an initial resistance mutation. However, in terms of fitness effects, it is irrelevant whether a second-site mutation occurs prior to, or after, the resistance mutation for which it compensates (although second-site mutations that are deleterious on a drug sensitive background are unlikely to segregate independently of the resistance mutation; Schrag et al., 1997). As such, I here refer to as compensatory any mutation that restores fitness costs imposed by a resistance mutation (**Figure 3**), regardless of when it arises.

In demonstrating the effects of genetic background on the fitness of resistance mutations, it is worth noting that laboratory measurements of the fitness of AMR mutations are almost always limited to a single genetic background: A resistance mutation is introduced onto a single “wild-type” background – typically a standard lab strain such as *E. coli* K-12 – and fitness is measured by competing this defined mutant against the wild-type control. However, a particular AMR mutation may or may not impose the same costs on different genetic backgrounds. In *Campylobacter jejuni*, costs associated with the *gyrA* C257T mutation, which confers fluoroquinolone resistance (FQ^R^), are strongly dependent on genetic background (Luo et al., 2005). On one genetic background, the FQ^R^ allele outcompetes the FQ^S^ allele in a chicken model of infection, whereas the FQ^S^ allele outcompetes the FQ^R^ allele on a different genetic background, indicating the presence of genetic modifiers – that is, compensatory mutations – elsewhere in the genome. Similar patterns, whereby the fitness effect of an AMR mutation varies with genetic background, have also been described for rifampicin resistance in *P. aeruginosa* (MacLean et al., 2010) and *Bacillus subtilis* (Cohan et al., 1994), and for clarithromycin resistance in *Helicobacter pylori* (Björkholm et al., 2001). The few available data suggest that genetic background can also influence the fitness of MGEs. For example, the conjugative plasmid RP1 was found to impose fitness costs in a laboratory strain, as well as in two clinical isolates, of *E. coli*, but not in two strains isolated from pig feces (Humphrey et al., 2012; see also Enne et al., 2004a; Starikova et al., 2013; Di Luca et al., 2017).

Compensatory evolution has been proposed as an important mechanism for the persistence of AMR strains in the absence of antibiotics (Andersson, 2003, 2006; Melnyk et al., 2015, but see MacLean and Vogwill, 2014 for a different perspective). *In vitro* selection experiments, whereby populations bearing a costly resistance mutation are passaged in a drug-free environment, have shown that compensatory evolution occurs readily in the laboratory for a wide range of resistance elements and bacterial species (for chromosomal mutations, see Schrag and Perrot, 1996; Bjorkman et al., 2000; Reynolds, 2000; Nagaev et al., 2001; Maisnier-Patin et al., 2002; Enne et al., 2004b; Kugelberg et al., 2005; Maisnier-Patin et al., 2007; Paulander et al., 2007; Brandis et al., 2012; Hall, 2013; Knopp and Andersson, 2015; Qi et al., 2016; for MGEs see Helling et al., 1981; Bouma and Lenski, 1988; McDermott et al., 1993; Dahlberg and Chao, 2003; Dionisio et al., 2005; Heuer et al., 2007; Starikova et al., 2013; San Millan et al., 2014b). Thus, for any given resistance mutation, there may be many available compensatory mutations. In some cases, compensation has been observed between pairs of resistance mutations for the same drug – for example, individually costly Rif^R^ mutations in *P. aeruginosa* can compensate for each other (Hall and MacLean, 2011, see also Rozen et al., 2007), leading to both increased fitness and increased drug resistance. Importantly, in *in vitro* compensation experiments, reversion of resistance mutations is rare relative to second-site mutations conferring fitness benefits (putative compensatory mutations; Hughes and Andersson, 2015), implying that resistant genotypes are likely to regain fitness in a drug-free environment without losing resistance.

The mechanisms underlying compensation have been described in several cases, and often involve restoring processes that are disrupted by the initial resistance mutation (for broader discussions of the mechanisms of epistasis see Boone et al., 2007; de Visser et al., 2011). Knopp and Andersson (2015), for example, investigated mechanisms of compensation in *ompCF* double mutants of *E. coli. ompC* and *ompF* encode outer membrane porins by which a number of antibiotics, including carbapenems, gain access to the periplasm. Mutations in these proteins confer modest- to high-levels of carbapenem resistance (fourfold for meropenem, 60-fold for ertapenem), but with an associated cost (∼15% in this study). After 250 generations of *in vitro* selection, fitness was recovered to between 89 and 99% of the wild-type, with carbapenem resistance maintained in some cases and lost in others. Subsequent analyses showed that compensation occurred via over-expression of the alternative porins PhoE or ChiP, which presumably replaced important physiological functions of OmpCF.

Restoration of normal cellular functions is also implicated in compensation for costs associated with disrupted transcription and translation. In many bacteria, mutations in *rpoB*, which encodes the β-subunit of RNA polymerase, are known to cause high levels of rifampicin resistance, typically with an associated fitness cost. In laboratory experiments using *Salmonella enterica*, compensation consistently occurs via mutations in *rpoA. rpoB*, and *rpoC* (Brandis et al., 2012), where *rpoA* and *rpoC* encode additional subunits of RNA polymerase. Structural considerations suggest that these compensatory mutations improve deleterious effects of the *rpoB* R529C mutation on rates of transcription, for example by improving RNA stability or by affecting the structure of the exit channel in which residue 529 is located (Brandis et al., 2012). Similarly, Schrag and Perrot (1996) found that protein elongation rates were reduced in streptomycin resistant *rpsL* mutants of *E. coli*, and that elongation rates were increased following compensatory evolution.

Less is known concerning the mechanisms underlying compensation for costs associated with plasmids and other MGEs. Several older studies suggested that compensatory mutations were to be found on the host chromosome, rather than on the plasmid (Helling et al., 1981; Bouma and Lenski, 1988). More recently, sequencing has corroborated these results in *P. aeruginosa* (San Millan et al., 2014b), where carriage of the costly plasmid pNUK73 was compensated following laboratory selection experiments. In six compensated clones, no mutations were found on pNUK73, but each carried a mutation in either of two chromosomal genes, one encoding a putative helicase, and the other encoding a putative protein kinase. Mutations in both genes were shown to reduce segregational loss of the plasmid, although the precise mechanism underlying this increased stability has yet to be elucidated.

While it is clear that compensatory evolution occurs readily in the laboratory, the clinical and epidemiological importance of compensation is still unclear. For example, in the absence of antibiotic, resistant strains may be outcompeted by resident sensitive strains before compensation occurs (MacLean and Vogwill, 2014, but see Gifford and MacLean, 2013). We simply have little data on the frequency of compensatory evolution in patient populations. Nonetheless, there is good evidence for clinically relevant compensatory evolution in rifampicin resistant strains of *Mycobacterium tuberculosis*. Mutations in *rpoA. rpoB*, and *rpoC*, similar to those implicated in compensatory evolution in *S. enterica*, have been identified in Rif^R^ – but not in Rif^S^ – clinical isolates of *M. tuberculosis* in several studies (Cohen et al., 2015; Coscolla et al., 2015), consistent with compensation in a clinical setting. Moreover, *rpoB* mutations bearing a high cost in *Salmonella* are more likely to be associated with putative compensatory mutations in *M. tuberculosis* (Brandis et al., 2015), again suggesting an important role for compensation. Overall, however, there is a need to further investigate the contribution of compensatory evolution to the persistence of AMR in clinical settings; this could be accomplished by screening clinical strains for compensatory mutations known from laboratory studies.

Interactions between AMR mutations and compensatory mutations are typically conceived of as sign epistatic, whereby the AMR mutation is deleterious on some backgrounds, but neutral or advantageous on others. Brandis et al. (2012), for example, measured growth rates for 10 mutations that compensate for fitness deficits in rifampicin resistant mutants of *S. enterica*. Each of these mutations, which were found in *rpoA* or *rpoC*, partially or completely restored growth defects caused by the *rpoB* R529C resistance mutation. However, in isolation, the compensatory mutations were either deleterious or had no effect on growth rates. These mutations were thus beneficial on the resistant background but not on the wild-type background, fulfilling the definition of sign epistasis. Interestingly, the persistence of a resistance mutation is promoted when compensatory mutations are deleterious on the drug sensitive background: reversion mutations are selected against, since reversion simply generates the drug sensitive background with the compensatory mutation (Schrag et al., 1997).

While there are a number of documented cases of sign epistasis between resistance mutations and compensatory mutations, there is a certain amount of ambiguity as to whether sign epistasis is a *necessary* condition for considering a mutation to be compensatory. For example, what are we to make of a candidate compensatory mutation that is equally beneficial on both resistant and susceptible backgrounds? Or of a mutation that is beneficial on resistant and susceptible backgrounds, but more beneficial on the resistant background (a case of positive epistasis)? Neither of these cases meets the strict definition of sign epistasis, but could nonetheless contribute to the persistence of AMR alleles in pathogen populations. It is not my intent to resolve this semantic issue here, but rather simply to point out the potential for conceptual ambiguity.

## Epistasis and the Evolution of Multi-Drug Resistance

Multi-drug resistance typically involves the accumulation of multiple resistance mutations and/or plasmid-borne resistance elements; alternatively, single mutations affecting efflux or permeability can cause MDR, but typically at lower levels than multiple-mutation driven MDR (reviewed in Chang et al., 2015). There is thus ample opportunity for genetic interactions between chromosomal mutations, between plasmids, or between chromosomal mutations and plasmids, in the evolution of MDR. The nature of such genetic interactions may be an important factor in the persistence of MDR strains. Consider, for example, a case where two individual resistance mutations are costly – that is, they are each less fit than susceptible competitors in the absence of drug. If the double mutant is less fit than either single mutant (no epistasis or negative epistasis), then it will be outcompeted by the single mutants and by susceptible strains. Alternatively, if the double mutant is more fit than at least one of the single mutants (positive sign epistasis), then it is expected to at least outcompete that single mutant. In the most extreme case, the double mutant may be as fit, or more fit, than susceptible competitors, in which case the MDR strain is expected to outcompete even susceptible strains in the absence of drug.

Studies across a range of bacterial species and resistance mechanisms have revealed widespread positive epistasis with respect to fitness in the absence of drug, including cases where the fitness of MDR strains equals or exceeds susceptible competitors (Trindade et al., 2009; Ward et al., 2009; Angst and Hall, 2013; Borrell et al., 2013; Durao et al., 2015). For example, Trindade et al. (2009) examined epistasis between mutations in *rpoB. rpsL* (streptomycin resistance), and *gyrA* (quinolone resistance) in *E. coli*. Epistasis was detected for more than half of the 103 double mutants, and 73% of cases of epistasis were positive, i.e., double mutant fitness was higher than expected. Double mutant fitness was equal to or greater than that of the isogenic susceptible control in six cases; these genotypes might be expected to be particularly common in clinical settings, at least insofar as laboratory fitness estimates are predictive of fitness in patients. Similarly, positive epistasis has been documented between chromosomal resistance mutations and conjugative plasmids (Silva et al., 2011), and between pairs of plasmids (San Millan et al., 2014a). By contrast, a recent study found that genes present on a chromosomally integrated MGEs were responsible for reductions in plasmid fitness in *P. aeruginosa* (San Millan et al., 2015); in this case, negative epistatic interactions might be expected to limit evolution by horizontal transfer.

Widespread positive epistasis between resistance mutations in different genes contrasts sharply with recent studies on intragenic epistasis, where negative epistasis predominates (Bank et al., 2015; Li et al., 2016; Puchta et al., 2016; Sarkisyan et al., 2016). Bank et al. (2015), for example, estimated fitness for more than 1000 double mutants (and their corresponding single mutants) of the yeast heat shock protein Hsp90. Almost half of the double mutants showed negative epistasis, while positive epistasis was observed in only 1.8% of cases. The reasons underlying this difference in the distribution of epistatic effects are unclear. For example, this distribution may differ for intragenic and extragenic mutation pairs, due to the nature of functional interactions between these mutations – consistent with this hypothesis, positive and negative epistasis are roughly equally prevalent for transposon insertions in *E. coli* (Elena and Lenski, 1997). Further empirical and theoretical work is needed to clarify this issue.

There is limited but compelling evidence that positive epistasis drives the evolution of MDR in clinical settings. Borrell et al. (2013) measured the fitness of 17 genotypes of *Mycobacterium smegmatis* resistant to both rifampicin and ofloxacin, as well as of the constituent single *rpoB* and *gyrA* mutants. Notably, the *gyrA* D94G mutation was associated with fitness increases for all five *rpoB* alleles that were tested, whereas the *gyrA* G88C allele was associated with reduced fitness. Correspondingly, in a sample of 151 MDR and XDR (extensively drug resistant) clinical isolates of *M. tuberculosis* (that are, by definition, resistant to both rifampicin and ofloxacin), 71 bore the D94G allele, while the G88C allele was present in only one. Together, these results suggest that positive epistatic interactions between *gyrA* D94G and *rpoB* mutations favor the survival of genotypes bearing D94G. Nonetheless, more data are needed on the distribution of resistance alleles in clinical isolates to make broad inferences about the impact of epistasis in maintaining MDR.

## Management of AMR: Clues From Epistasis

As discussed in the previous two sections, laboratory experiments – and to a lesser extent studies of clinical isolates – suggest important roles for epistasis in maintaining single- and multi-drug resistance, thus increasing the challenges associated with tackling AMR. More optimistically, there are opportunities to leverage epistasis in the management of AMR.

Knowledge of the epistatic interactions of resistance mutations should help to inform decisions as to which drug combinations to deploy in clinical settings. Drug combinations are often chosen to maximize the rate of clearance. In particular, synergistic drug combinations – whereby the combined inhibitory effect of two drugs is stronger than expected – are often preferred for their ability to clear infections. However, the use of synergistic drug combinations also increases selection on resistance alleles, since resistance to one drug also alleviates the synergistic effects. Consequently, the use of synergistic drug combinations can *promote* the evolution of resistance, at least under some conditions (Hegreness et al., 2008; Michel et al., 2008; Torella et al., 2010). Thus, it has been suggested that the primary goal of multi-drug therapy should be to *slow* or *prevent* the evolution of resistance (Chait et al., 2007; Yeh et al., 2009; Imamovic and Sommer, 2013; Palmer and Kishony, 2013; Bollenbach, 2015; Baym et al., 2016), rather than to clear infections quickly.

Knowledge of the epistatic interactions of resistance mutations can and should inform such efforts to minimize the evolution of AMR via multi-drug therapy. There are several ways in which epistasis is relevant. First, negative epistasis between resistance mutations might be leveraged to counteract the evolution of MDR in clinical populations. In some cases, pairs of resistance mutations interact negatively – that is, the fitness of the double mutant is *lower* than expected given the fitness of the single mutants. As such, genotypes resistant to both drugs have highly reduced fitness, and are expected to be outcompeted by single-resistant and susceptible genotypes. Thus, it has been proposed that we should employ pairs of antibiotics for which there is negative epistasis between resistance mutations (Muller et al., 2013).

Cases of negative epistasis have indeed been documented for particular pairs of resistance alleles. For example, Trindade et al. (2009) found strongly negative epistasis between K43T mutations in *rpsL* and D87G mutations in *gyrA* in *E. coli*. Unfortunately, other pairs of quinolone- and streptomycin-resistance conferring mutations show no epistasis or positive epistasis, e.g., *rpsL* K43R and *gyrA* D87G. Thus, in clinical samples resistant to both streptomycin and quinolones, we might expect to see an excess of K43R/D87G genotypes and a deficit of K43T/D87G genotypes. However, an overall reduction in the frequency of doubly-resistant genotypes is not necessarily expected. Indeed, this example highlights the fact that reducing the prevalence of MDR genotypes requires negative interactions between most or all possible resistance mutations at two (or more) loci. If some pairs of resistance mutations have relatively high fitness, then multi-drug treatment will simply select for those pairs. Such systematic negative epistasis between sets of resistance mutations has not been documented; there instead seems to be a great deal of allele-specificity in genetic interactions (Trindade et al., 2009). Nonetheless, genetic interactions between AMR mutations have simply not been investigated for the majority of drugs, nor in the majority of pathogens, so we cannot rule out the plausibility of the approach.

Another approach to counteracting the evolution of AMR is to take advantage of the *collateral drug sensitivity* imposed by some resistance mutations (e.g., Imamovic and Sommer, 2013; Lazar et al., 2013; Munck et al., 2014; Oz et al., 2014). Certain resistance mutations confer increased sensitivity to other drugs. Such mutations are, therefore, pleiotropic, insofar as they affect traits other than the one for which they were selected. For example, aminoglycoside resistance is frequently accompanied by increased sensitivity to multiple drug classes, including quinolones, tetracyclines, and β-lactams (Imamovic and Sommer, 2013; Lazar et al., 2013; Oz et al., 2014). Reductions in proton-motive force (PMF), which confer low-level aminoglycoside resistance, are thought to underlie this collateral sensitivity (Lazar et al., 2013). The existence of collateral sensitivity suggests the use of co-dosing regimens informed by knowledge of collateral sensitivities – co-dosing between an aminoglycoside and a quinolone might, for example, slow the emergence of aminoglycoside resistance. Laboratory selection experiments confirm the potential to slow the evolution of resistance by leveraging collateral sensitivity (Kim et al., 2014). Populations of *Staphylococcus aureus* were evolved under mixed- or alternating treatments of pairs of drugs. Resistance evolution was generally slowed compared to single-drug controls, and patterns of collateral sensitivity predicted the outcomes. For example, most single-step trimethoprim-resistant mutants of *S. aureus* were collaterally sensitive to ciprofloxacin. Correspondingly, the acquisition of trimethoprim resistance was slowed in mixed trimethoprim-ciprofloxacin treatments.

Typically, collateral sensitivities are identified using chemical-genetic approaches, whereby MICs are determined for a wide range of known antibiotics against a set of AMR genotypes (Imamovic and Sommer, 2013; Lazar et al., 2013). Similarly, novel screening approaches have been developed to identify microbially derived compounds that are particularly effective against AMR strains (Chait et al., 2010). Genetic interaction screens may be a useful complement in identifying collateral sensitivities. For example, if a particular AMR mutant were to show negative interactions with knockouts in a number of cell wall genes, then this would suggest that drugs targeting the cell wall (e.g., β-lactams, fosfomycin) might select against that AMR mutation. Moreover, such screens might identify novel drug targets that interact negatively with an AMR mutation, with no currently available antibiotic for that target. To date, large-scale genetic interaction screens have not included many AMR mutations; chromosomal AMR mutations are often point mutations in essential genes, and essential genes are by definition not included in the knockout collections employed in most large-scale strategies. There is thus a need to expand large-scale screens to include point mutants as “baits,” in order to uncover mutations that interact with the specific mutations underlying AMR. Indeed, the success of genetic interaction screens in identifying novel drug targets specific to cancer cells provides promise for this approach in combatting AMR (Hartwell et al., 1997; Kaelin, 2005; Shaheen et al., 2011).

While these examples suggest that knowledge of the genetic interactions of AMR mutations can inform efforts to manage resistance, a word of warning is in order. These approaches all aim to maximize the costs of resistance, either in the absence of drugs, or, in the case of collateral sensitivity, in the presence of specific antibiotics. While maximization of the cost of resistance is clearly an admirable goal, it too has its drawbacks. As illustrated earlier in this manuscript, compensatory evolution occurs rapidly and easily; moreover, low-fitness (high cost) genotypes compensate more quickly than higher-fitness genotypes (Moore et al., 2000; Gifford et al., 2011). Thus, unless high-cost genotypes are eliminated very quickly from a population, we should expect that they too will persist due to compensatory evolution.

## Conclusion

Epistasis is a widespread phenomenon, with 10s–100s of 1000s of genetic interactions identified in large-scale screens in model microbes (Butland et al., 2008; Typas et al., 2008; Costanzo et al., 2010). The epistatic interactions of AMR mutations may play important roles in the persistence of resistance, via compensatory evolution, and in the evolution of MDR, due to positive epistasis between different resistance mutations. Moreover, an understanding of the epistatic interactions of AMR mutations would be helpful in developing strategies – and therapeutic agents – for mitigating the evolution of resistance.

Given the importance of epistasis in the evolution and management of AMR, broader efforts are needed to fully characterize the genetic interactions of AMR mutations. Large-scale screens measuring the fitness of AMR mutants in combination with genome-wide knockout libraries, or with over-expression constructs, will be valuable tools in this effort. Moreover, laboratory selection experiments can yield important information on epistatic interactions. Here, replicate populations are passaged for 10s–1000s of generations under defined conditions in the laboratory, and whole-genome sequencing can be used to identify adaptive mutations that have arisen over the course of selection. Epistasis can be inferred from patterns of co-occurrence, or lack thereof, between mutations. For example, following 150 generations of selection in sub-inhibitory concentrations of ciprofloxacin, we found that a given genotype would bear a mutation in either *gyrA* or *gyrB* (the genes encoding DNA gyrase, the target of ciprofloxacin), but never in both (Wong and Seguin, 2015). This result suggests that, under the selection conditions, mutations in *gyrA* or *gyrB* were individually adaptive, but that double mutants gained no additional benefit – indicating negative epistasis between mutations in these two genes.

There are relatively few data concerning the genetic interactions of plasmids or other MGEs. The available evidence suggests that certain broad patterns may be shared between chromosomal mutations and plasmids. Fitness costs are associated with both, although both chromosomal mutations and MGEs may be cost-free or even advantageous in the absence of drug. In both cases, positive epistasis may play an important role in reducing fitness costs where they are present. Nonetheless, given the critical importance of horizontally transferred resistance, for example for β-lactams and aminoglycosides (e.g., Davies, 1994), much more effort is required to characterize the genetic interactions of MGEs. Moreover, theoretical and empirical investigations will help to determine the contribution of epistasis to the persistence of MGEs, as opposed to other factors such as co-selection or spatially variable selection.

Similarly, there are few data concerning the genetic interactions of efflux mutations, despite the importance of efflux in MDR in a variety of pathogens (Vila et al., 2007; Vila and Martinez, 2008; Hirsch and Tam, 2010; Wong and Kassen, 2011). Mutations that increase efflux pump expression are sometimes, but not always, costly in *in vitro* assays (Alonso et al., 2004; Stickland et al., 2010; Olivares et al., 2012), and have been shown to negatively impact virulence in *P. aeruginosa* (Olivares et al., 2012) and *Stenotrophomonas maltophilia* (Alonso et al., 2004). However, the interactions of efflux mutations with other resistance mutations, as well as mechanisms of compensation for costly efflux mutations, remain to be investigated.

Finally, as suggested throughout this review, more work is needed to determine the impact of epistasis in clinical pathogen populations. With the advent of rapid, cost-effective whole-genome sequencing, we can search the genomes of clinical isolates for compensatory mutations identified in the laboratory (Cohen et al., 2015; Coscolla et al., 2015). Similarly, we might predict that pairs of resistance elements that show positive epistasis should be over-represented in clinical MDR strains. Such data will help us to determine the importance of epistasis in the evolution of clinical pathogen populations, and will in turn inform efforts to mitigate the evolution of resistance.

## Author Contributions

AW conceived of and wrote this manuscript.

## Conflict of Interest Statement

The author declares that the research was conducted in the absence of any commercial or financial relationships that could be construed as a potential conflict of interest.
